# Laparoscopy in urology

**DOI:** 10.4103/0972-9941.19260

**Published:** 2005-10

**Authors:** Ashok K. Hemal, Rajeev Kumar

**Affiliations:** Department of Urology, All India Institute of Medical Sciences, New Delhi, India

Progress in most fields of surgery and in Urology in particular has been marked by an increasing use of minimal access surgery. The advantages of minimal access techniques are legion and do not need repetition. What does need reminding is the fact that the surgery is only as good as the surgeon performing it. Increasing public awareness and easier access to information through literature and the internet have made most patients aware of their options and has fueled the demand for laparoscopy. It is routine to get patients who will question your advice of open surgery and reasons for not offering minimal invasive or “laser” surgery as it is known in lay language.

This knowledge is healthy and improves the patient-doctor communication. However, it has also led to a situation where open surgery is considered “infradig” and a surgeon may be considered incompetent if he does not offer laparoscopy to his patient. Consequently, surgeons may be tempted to attempt something that they actually are not confident doing or should not be doing.

The articles in this special issue of the *Journal of Minimal Access Surgery* have been tailored to address these particular problems in urology. Each article deals specifically with the indications and contraindications of each procedure. It is important that patient selection be meticulous if complications are to be avoided. The articles then describe the basic technique of the procedure including common variants followed by results.

The articles do not aim to make expert laparoscopists out of the readers. They have been written to serve as guides in planning a procedure. Laparoscopic urology has a steep learning curve with significant potential for complications. Even today, it is a subspecialty of urology and these surgeries are being performed by dedicated urologic surgeons. When we talk of laparoscopic uro-oncology, the bar becomes even higher because of the need to ensure not only a safe surgery but also one that has oncological and functional results comparable to open surgery. Lest we may do more harm to our patients than benefit, laparoscopic urologic surgery should be performed only by surgeons adept at the open version of the procedure with appropriate training and experience.

The editors are grateful to the authors who have spent valuable time in writing these articles. These authors are among the leaders in their field and have considerable personal experience in the procedure that they describe. The manuscripts contain a wealth of knowledge and tips that only masters can provide.

We are also grateful to the editors and publisher of the *Journal of Minimal Access Surgery* who have given us the opportunity to compile this special issue. It clearly highlights the importance that laparoscopic urology has in today's surgical practice.

